# Pendelluft diagnosed from ventilator weaning indexes obtained through bioelectrical impedance tomography: a case report: *Pendelluft* diagnosticado através de índices de desmame ventilatório obtidos pela tomografia de bioimpedância elétrica: um relato de caso

**DOI:** 10.1590/1516-3180.2016.025514102016

**Published:** 2017-04-03

**Authors:** Fabiana Aparecida Lopes, Lidiane Andrade Monteiro de Souza, Juliana Tavares Neves Bernardi, Carlos Eduardo Rocha, Luciana Castilho de Figueiredo, Ana Paula Ragonete dos Anjos Agostini, Desanka Dragosavac, Daniela Cristina dos Santos Faez

**Affiliations:** I BSc. Physiotherapist at the Adult Intensive Care Unit, Universidade Estadual de Campinas (UNICAMP), Campinas (SP), Brazil.; II MSc, PhD. Physiotherapist at Adult Intensive Care Unit, Hospital das Clínicas, and Supervisor of Chest Physiotherapy Training Course, Adult Intensive Care Unit, Universidade Estadual de Campinas (UNICAMP), Campinas (SP), Brazil.; III MSc, PhD. Physiotherapist, Faculdade de Ciências Médicas, Universidade Estadual de Campinas (FCM-UNICAMP), Campinas (SP), Brazil.; IV MD, PhD. Coordinator, Adult Intensive Care Unit, Hospital das Clínicas, Universidade Estadual de Campinas (UNICAMP), Campinas (SP), Brazil.; V MSc. Physiotherapist, Faculdade de Ciências Médicas, Universidade Estadual de Campinas (FCM-UNICAMP), Campinas (SP), Brazil.

**Keywords:** Ventilator weaning, Respiration, artificial, Intensive care unit, Thoracic surgery, Acute lung injury

## Abstract

**CONTEXT:**

Today, through major technological advances in diagnostic resources within medicine, evaluation and monitoring of clinical parameters at the patient’s bedside in intensive care units (ICUs) has become possible.

**CASE REPORT:**

This case report presents results and interpretations from predictive mechanical ventilation weaning indexes obtained through monitoring using chest electrical bioimpedance tomography. These indexes included maximum inspiratory pressure, maximum expiratory pressure, shallow breathing index and spontaneous breathing test. These were correlated with variations in tidal volume variables, respiratory rate, mean arterial pressure and peripheral oxygen saturation. Regarding the air distribution behavior in the pulmonary parenchyma, the patient showed the pendelluft phenomenon. Pendelluft occurs due to the time constant (product of the airways resistance and compliance) asymmetry between adjacent lung.

**CONCLUSION:**

Bioelectrical impedance tomography can help in weaning from mechanical ventilation, as in the case presented here. Pendelluft was defined as a limitation during the weaning tests.

## RESUMO

**CONTEXTO:**

Atualmente, com o grande avanço tecnológico em recursos para diagnósticos em medicina, a avaliação e a monitorização de parâmetros clínicos à beira leito de paciente em unidade de terapia intensiva (UTI) se tornou possível.

**RELATO DE CASO:**

Neste relato de caso, apresentam-se os resultados e a interpretação de índices preditivos de desmame da ventilação mecânica obtidos pela tomografia de bioimpedância elétrica torácica. Esses índices incluíram a pressão inspiratória máxima, pressão expiratória máxima, índice de respiração superficial e teste de respiração espontânea. Estes estavam correlacionados com as variações de volume corrente, frequência respiratória, pressão arterial média e saturação periférica de oxigênio. Quanto ao comportamento da distribuição de ar no parênquima pulmonar, o paciente apresentou o fenômeno *pendelluft*. O *pendelluft* ocorre dado pela constante de tempo (produto da resistência e complacência das vias aéreas) de forma assimétrica entre as unidades pulmonares adjacentes.

**CONCLUSÃO:**

A tomografia de bioimpedância pode auxiliar no desmame da ventilação mecânica, como no caso apresentado. *Pendelluft* foi definido como limitação durante a execução dos testes para desmame.

## INTRODUCTION

Today, through major technological advances in diagnostic resources within medicine, evaluation and monitoring of clinical parameters at the patient’s bedside in intensive care units (ICUs) has become possible. Bioelectrical impedance tomography on these parameters is one example of these advances. It uses high-frequency electrical signals at low intensity to provide imaging of lung mechanics in real time. These signals are obtained by fixing a strap containing electrodes around the patient’s chest, to capture the intensity and frequency of the electric current that is propagated around the chest, between the electrodes. It is a noninvasive technique that does not use any type of radiation. It constitutes an innovation within interpretation of pulmonary mechanics.^1^

The predictive indexes for withdrawing patients from mechanical ventilation include the rapid shallow breathing index (RSBI), maximum inspiratory pressure (PImax) and maximum expiratory pressure (PEmax). The 2013 Brazilian guidelines for mechanical ventilation state that these indexes contribute towards decision-making in cases in which weaning is considered difficult. Thus, the decision-making for referring patients for a spontaneous breathing test (SBT) or for extubation does not rely on a single instrument. Use of these indexes may lead to shorter duration of mechanical ventilation.^2^^,^^3^

Use of chest electrical impedance tomography (EIT) at the bedside for patients undergoing a mechanical ventilation weaning process may be an important tool for aiding in this process. EIT takes into account important variables such as tidal volume, the degree of collapse of recruitable alveoli and the degree of alveolar distention. This test is based on differences in electrical properties generated by changes to air content in small regions of the lung, which create impedance between these regions. The pixels generated by the display image represented the percentage change in local impedance, relative to a reference that was obtained at the beginning of the image acquisition. Therefore, the dynamic images shown on the chest EIT monitor represent real-time local air changes during ventilation. At locations where variations in the air within the alveoli occur, the color of the image generated changes on a scale ranging from dark blue (less aeration) to light blue (greater aeration). Grey images represent regions in which there was no change of aeration.^4^^,^^5^

Pendelluft is a phenomenon that constitute a new mechanism of lung injury induced by mechanical ventilation. The overstretch that is observed in the dependent lung may cause a hidden injury point, that cannot be detected and thus is overlooked when conventional monitoring is used. Pendelluft can be defined as the air circulation within the lung parenchyma, in non-dependent and dependent areas, when there is no overall change in lung volume. Traditionally, it was believed that contraction of the diaphragm would decrease the pleural pressure uniformly, by the same amount at all points on the lung surface, so as to create a uniform increase in transpulmonary pressure. Pendelluft occurs because, in contrast to the normal lung, the injured lung does not show uniform fluid distribution behavior. Instead, transmission of local changes in pleural pressure is heterogeneous.^6^

Therefore, the objective of this study was to report on lung mechanics behavior, as shown by predictive weaning indexes obtained through bioelectrical impedance and by spontaneous breathing tests, in a patient with mitral valve disease who underwent valve replacement and prolonged weaning.

## CASE REPORT

The patient was a 65-year-old female with a history of surgical replacement of the mitral valve by a bioprosthesis 14 years earlier. Her personal history included valvular heart disease, atrial fibrillation, congestive heart failure (functional class IV) and hypertension.

At the time of admission to the clinical hospital of the University of Campinas on July 3, 2015, the patient had had symptoms of progressive dyspnea for the preceding six months. This was also associated with paroxysmal nocturnal dyspnea, orthopnea and lower limb edema. Therefore, the patient had been referred for evaluation of valve dysfunction and surgical assessment.

In the initial clinical evaluation, an echocardiogram was performed on July 6, 2015, which showed increased volume of the left chambers, presence of the biological mitral prosthesis with stenosis and moderate regurgitation, pulmonary hypertension with systolic pulmonary artery depression values of 78 mmHg and left-ventricle ejection fraction of 58% through Simpson’s method. Therefore, the patient was admitted to the hospital ward.

On July 7, 2015, her condition evolved with a productive cough and crackling and wheezing observed through auscultation. Pneumonia was diagnosed, which was treated with antibiotic therapy. The patient made continuous use of diuretic and antihypertensive drugs.

After 19 days of hospitalization, the patient underwent surgery for replacement of the bioprosthetic mitral valve with a mechanical prosthesis and also underwent tricuspid valve plasty. The procedure was performed under extracorporeal circulation, with a duration of 124 minutes. There were 46 minutes of myocardial ischemia and 92 minutes of aortic clamping without blood transfusions. No intraoperative events were noted. After surgery, the patient was transferred to the intensive care unit without sedation, with mechanical ventilation using dosages of dopamine diuretic. The patient was extubated in the immediate postoperative period and she started using a Venturi oxygen mask, receiving an inspired oxygen fraction (FiO_2_) of 50%.

**Figure 1:**
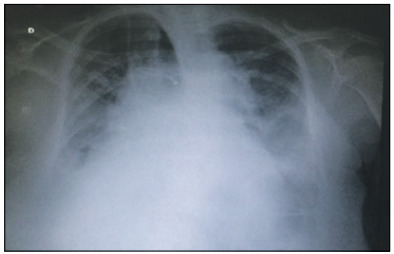
Chest X-ray in posterior-anterior view (April 24, 2015).

Two days after the operation, the patient developed worsening of symptoms. This included tachypnea, decreased peripheral oxygen saturation (SpO_2_) and significant radiological worsening, with diffuse infiltrates, signs of pulmonary congestion and opacification of the costophrenic angle of the breasts, as shown in Figure 1.

She also presented oliguria and it was necessary to administer furosemide intermittently. Noninvasive mechanical ventilation (NIV) was used for an intermittent period, but with little improvement of the tachypnea.

Since there was no improvement of respiratory symptoms, even through using NIV, it was decided on August 2, 2015, to perform an intubation procedure and start continuous sedation for better respiratory management. After the clinical signs had become stable, the weaning process was started.

At the minimum mechanical ventilation parameters, i.e. spontaneous mode with FiO_2_ of 40%, positive end-expiratory pressure (PEEP) of 4 cmH_2_0 and tidal volume of 5 ml/kg, the patient did not tolerate procedures to produce predictive indexes for withdrawal of mechanical ventilation. She showed signs suggestive of respiratory failure, according to the 2013 Brazilian guidelines for mechanical ventilation. A chest computed tomography (CT) scan was then performed, which revealed the presence of extensive bilateral pleural effusion at the bases of the lungs, and more evidently in the right lung. To relieve this, it was decided to perform thoracentesis, with removal of 1300 ml of citric fluid.

To evaluate the lung mechanics, respiratory monitoring through a scanner by means of bioelectrical impedance analysis was chosen (Timpel Enlight 1800, São Paulo, Brazil). An EIT electrode belt with 16 electrodes was placed around the thorax at the level of the fifth intercostal space, and one reference electrode was also placed on the patient. A tidal image was calculated as the difference between the EIT images at end-inspiration and end-expiration for one tidal breath, which represents the regional distribution of tidal volume (the tidal variation of impedance). Thus, the predictive indexes for success or failure in withdrawing mechanical ventilation and the spontaneous breathing test (SBT) using a T piece were determined and could then be evaluated by means of tomography.

**Graph 1:**
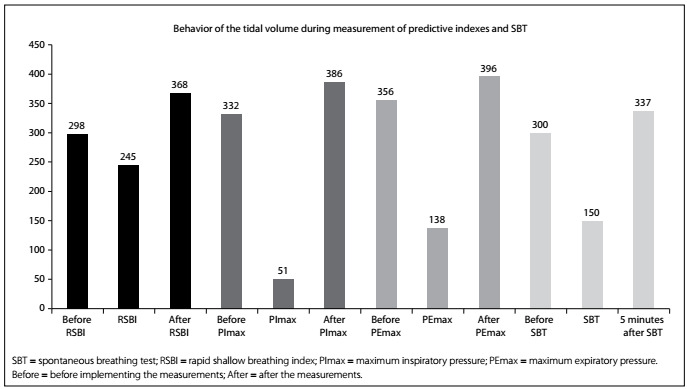
Behavior of the variation of tidal volume during measurement of the predictive indexes and spontaneous breathing test (SBT) by means of electrical bioimpedance tomography.

**Figure 2:**
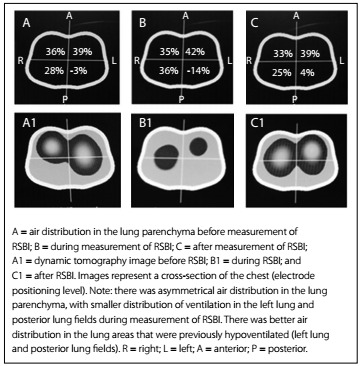
Behavior of the air distribution in the lung parenchyma before, during and after measurement of the rapid shallow breathing index (RSBI).

**Figure 3:**
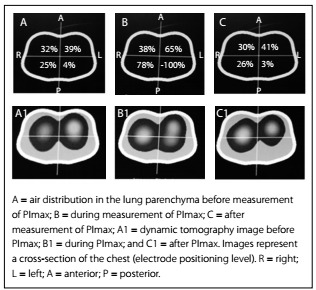
Behavior of the air distribution in the lung parenchyma before, during and after measurement of the maximum inspiratory pressure (PImax).

**Figure 4:**
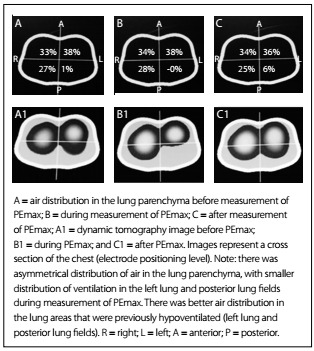
Behavior of the air distribution in the lung parenchyma before, during and after measurement of the maximum expiratory pressure (PEmax).

**Figure 5:**
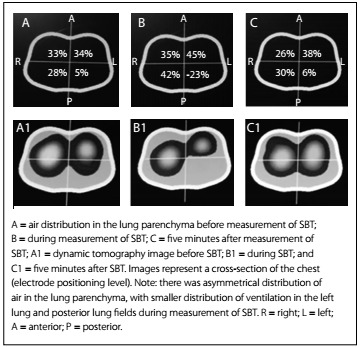
Behavior of the air distribution in the lung parenchyma before, during and five minutes after measurement of the spontaneous breathing test (SBT).

The results regarding the variation of heart rate (HR), respiratory frequency (RF), peripheral oxygen saturation (SpO_2_) and mean arterial pressure (MAP) are shown in Table 1. Any significant variations in tidal volume and in the distribution of lung parenchyma could be checked through determining predictive indexes (RSBI, PImax and PEmax) from electrical bioimpedance tomography images and through determining spontaneous breathing. The behavior of changes in tidal volume at the time of determining the predictive indices and SBT are illustrated in Graph 1. The changes in the distribution of air in the lung parenchyma are illustrated in Figures 2, 3, 4 and 5. These were obtained from the scanner screen through bioimpedance at the following times: before, during and after determining the predictive indices and before and 5 minutes after completion of the spontaneous breathing test.

**Table 1:**
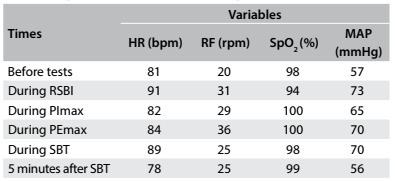
Variation of heart rate (HR), respiratory frequency (RF), peripheral oxygen saturation (SpO_2_) and mean arterial pressure (MAP), before measuring the predictive indexes, during measurement of the predictive indexes and five minutes after performing the spontaneous breathing test (SBT)

bpm = beats per minute; rpm = respiration per minute; % = percentage; MAP = mean arterial pressure; mmHg = millimeters of mercury; RSBI = rapid shallow breathing index; PImax = maximum inspiratory pressure; PEmax = maximum expiratory pressure; SBT = spontaneous breathing test.

Importantly, the patient developed a high response to atrial fibrillation at the time of the spontaneous breathing test, without hemodynamic repercussions. Five minutes after this test was started, the patient showed signs of respiratory failure. This requiring discontinuation of the test and return to mechanical ventilation in spontaneous mode, with the minimum parameters already described above. It was also observed that after the predictive indexes had been determined, there was better distribution of air in the lung parenchyma. This was shown through the positive graphic percentages displayed on the scanner screen (Figures 2, 3, 4 and 5).

The behavior of the air distribution within the lung parenchyma before, during and after determining the RSBI can be seen in the ventilation map distribution of 64%, 71% and 58% in the right lung and 36%, 28%, 43% in the left lung, as shown in Figure 2.

From these findings and because the weaning process failed for a second time, it was decided during a clinical visit and discussion of the case to perform tracheostomy. This was done on August 11, 2015. On the days following this, progressive reduction of ventilatory parameters was observed and the weaning process was again started, using intermittent mist in a T piece. The patientprogressed satisfactorily and continued to receive continuous nebulization. After a few days, the plastic tracheostomy tube was exchanged for a metal one.

## DISCUSSION

Changes in lung function that occur subsequent to cardiac surgery using extracorporeal circulation are secondary to reactions to use of heparin and comprise protamine complex, edema, congestion, lung injury and microatelectasis. In most cases, mechanical ventilation is absent during extracorporeal circulation. This, together with the inflammatory response due to surgical trauma, leads to changes in respiratory function, consistent with those presented in cases of acute respiratory distress syndrome.^7^^,^^8^

Rodrigues et al. suggested that during and after cardiac surgery, transient dysfunction of gas exchange (TDGE) is evident to varying degrees. Patients with preoperative hypertension and cardiogenic shock presented an association with occurrence of postoperative TDGE. During the postoperative period, presence of pneumonia, need for renal replacement therapy, need for blood therapy and presence of cardiac arrhythmias were correlated with the appearance of a degree of TDGE, thus indicating a risk factor for reintubation.^9^

The case that Rodrigues et al. published consisted of a patient who was reintubated on the second day after cardiac surgery.^9^ This patient presented a high response of atrial fibrillation while performing a SBT using a T piece without hemodynamic repercussions. Five minutes after the SBT started, the patient showed signs of respiratory failure and the SBT had to be discontinued. There was a second failure in withdrawing mechanical ventilation and conducting tracheostomy.

The images of Figures 2, 3, 4 and 5 were obtained in determining rapid shallow breathing index superficial speed breathing index (RSBI), PImax, PEmax and SBT. They show variations in the distribution of air in the lung parenchyma. The behavior exhibited is compatible with the pendelluft effect.

Greenblatt et al. showed that presence of the pendelluft effect may arise due to use of mechanical ventilation in spontaneous mode in cases in which spontaneous breathing efforts are detected, especially when the respiratory rate is high.^10^^,^^11^

Yoshida et al. conducted an experimental clinical study in which acute lung injury was induced in seven pigs, with the objective of making comparisons with respiratory monitoring data from a clinical case of a male patient who underwent surgery for coronary revascularization. They observed that when the seven pigs with acute lung injury were under mechanical ventilation without showing spontaneous ventilation effort, there was simultaneous inflation in different areas of their lungs. However, when the pigs showed spontaneous inspiratory efforts during mechanical ventilation, initial inflation of the dependent lung and simultaneous deflation could be observed. Despite these authors’ findings, it seems that the pendelluft phenomenon can increase alveolar recruitment in areas of atelectasis that are selectively dependent on inflation, and that the degree of pendelluft is proportional to the intensity of spontaneous effort on mechanical ventilation. These findings corroborate what was found in the present case study.^12^

Greenblatt et al. considered that pendelluft may be more evident in diseases with a heterogeneous pattern of injury and major changes in respiratory mechanics, with changes in strength and in lung compliance. In the present case study, the chest X-ray suggested that the heterogeneous behavior described by these authors also occurred here.^10^

Recently, Yoshida et al. recommend that it would be important to balance muscle paralysis in relation to maintenance of spontaneous breathing during mechanical ventilation for patients with acute respiratory distress syndrome network (ARDSnet). This would depend on the severity of ARDSnet, its evolution phase and its respiratory demands. In the early phase of severe ARDSnet, partial ventilatory support to promote spontaneous breathing should be avoided. Paralysis of the diaphragm muscle may be effective for preventing pendelluft. In situations of less severity of ARDSnet, and after a short period of diaphragm muscle paralysis in cases of severe ARDSnet, spontaneous breathing should be facilitated by means of partial mechanical ventilatory support. This would prevent large spontaneous respiratory efforts.^13^

The finding of pendelluft can be determined through noninvasive monitoring conducted by means of electrical bioimpedance tomography, without exposing the patient to the invasive measures of other forms of monitoring. Furthermore, this monitoring can make a substantial contribution to research, thus facilitating implementation of a series of studies that together can assist in understanding abnormal physiology. This would include investigating the regional heterogeneity of ventilation and factors associated with pendelluft. A few studies have been conducted on pendelluft diagnosed during weaning. The results from a systematic search in the main databases in the literature are presented in Table 2. Electrical bioimpedance tomography is simple to be performed at the bedside and has the potential to provide better understanding of the pathophysiology of a variety of lung disorders resulting from mechanical ventilation.^14^^,^^15^

**Table 2:**
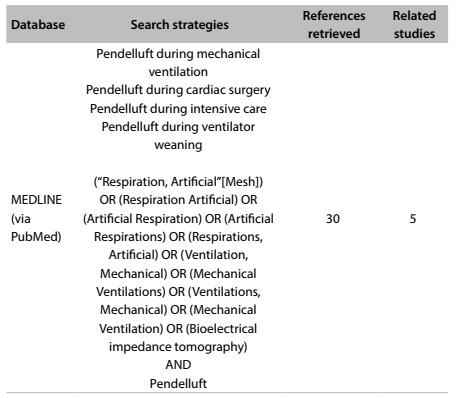
Search of the literature in medical databases on October 17, 2016, for cases of pendelluft that have been diagnosed

## CONCLUSION

Electrical bioimpedance tomography can contribute towards interpreting failures of withdrawal of ventilatory support and towards monitoring the side effects caused by mechanical ventilation, especially in patients with heterogeneous patterns of changes to respiratory mechanics. The findings from predictive indexes relating to withdrawal of mechanical ventilation might enable better distribution of air in the lung parenchyma and increased lung homogenization.

It should also be noted that after the predictive indexes had been determined, the tidal volume did not return to its initial value. This effect was caused by alveolar recruitment through the increased respiratory effort during testing.

Electrical bioimpedance tomography can help in weaning patients off mechanical ventilation, as in the case presented here. The limitation of pendelluft while tests on weaning were being performed was defined.

Further studies should be conducted to investigate whether the alveolar recruitment achieved after conducting spontaneous effort, at the time of determining the predictive indices, was maintained after long reconnection to mechanical ventilation.
